# Association between *MGMT* Promoter Methylation and Non-Small Cell Lung Cancer: A Meta-Analysis

**DOI:** 10.1371/journal.pone.0072633

**Published:** 2013-09-26

**Authors:** Changmei Gu, Jiachun Lu, Tianpen Cui, Cheng Lu, Hao Shi, Wenmao Xu, Xueli Yuan, Xiaobo Yang, Yangxin Huang, Meixia Lu

**Affiliations:** 1 Department of Epidemiology and Biostatistics and the Ministry of Education Key Laboratory of Environment and Health, School of Public Health, Tongji Medical College, Huazhong University of Science and Technology, Wuhan, Hubei, China; 2 The Institute for Chemical Carcinogenesis, the State Key Laboratory of Respiratory Disease, School of Public Health, Guangzhou Medical University, Guangzhou, Guangdong, China; 3 Departments of Clinical Laboratory, Wuhan First Hospital, Wuhan, Hubei, China; 4 Department of Anatomy, Medical College of Nanchang University, Nanchang, Jiangxi, China; 5 Department of Occupational Health and Environmental Health, School of Public Health, Guangxi Medical University, Nanning, Guangxi, China; 6 Department of Epidemiology and Biostatistics, College of Public Health, University of South Florida, Tampa, Florida, United States of America; University of Torino, Italy

## Abstract

**Background:**

O^6^-methylguanine-DNA methyltransferase (MGMT) is one of most important DNA repair enzyme against common carcinogens such as alkylate and tobacco. Aberrant promoter methylation of the gene is frequently observed in non-small cell lung cancer (NSCLC). However, the importance of epigenetic inactivation of the gene in NSCLC published in the literature showed inconsistence. We quantified the association between *MGMT* promoter methylation and NSCLC using a meta-analysis method.

**Methods:**

We systematically reviewed studies of *MGMT* promoter methylation and NSCLC in PubMed, EMBASE, Ovid, ISI Web of Science, Elsevier and CNKI databases and quantified the association between *MGMT* promoter methylation and NSCLC using meta-analysis method. Odds ratio (OR) and corresponding 95% confidence interval (CI) were calculated to evaluate the strength of association. Potential sources of heterogeneity were assessed by subgroup analysis and meta-regression.

**Results:**

A total of 18 studies from 2001 to 2011, with 1, 160 tumor tissues and 970 controls, were involved in the meta-analysis. The frequencies of *MGMT* promote methylation ranged from 1.5% to 70.0% (median, 26.1%) in NSCLC tissue and 0.0% to 55.0% (median, 2.4%) in non-cancerous control, respectively. The summary of OR was 4.43 (95% CI: 2.85, 6.89) in the random-effects model. With stratification by potential source of heterogeneity, the OR was 20.45 (95% CI: 5.83, 71.73) in heterogeneous control subgroup, while it was 4.16 (95% CI: 3.02, 5.72) in the autologous control subgroup. The OR was 5.31 (95% CI: 3.00, 9.41) in MSP subgroup and 3.06 (95% CI: 1.75, 5.33) in Q-MSP subgroup.

**Conclusion:**

This meta-analysis identified a strong association between methylation of *MGMT* gene and NSCLC. Prospective studies should be required to confirm the results in the future.

## Introduction

Lung cancer is the leading cause of global cancer deaths in recent decades [1]. Human lung cancer contains two histological types, small-cell lung cancer (SCLC) and non-small lung cancer (NSCLC). The latter comprises the majority of lung cancer and has an increasing incidence and mortality in the last two decades worldwide. DNA methylation is an epigenetic modification of the genome and methylation associated with silencing can affect genes expression in cellular pathways [2]. The epigenetic alterations are early and frequent events occurred in carcinogenesis. O^6^-methylguanine-DNA methyl-transferase (MGMT) is a DNA damage reversal protein against DNA adduct formation of carcinogens [3,4]. It can protect cells from the carcinogenic effects of alkylating agents by removing adducts from the O^6^ position of guanine [5]. Therefore, the repair capacity of the MGMT protein helps decrease the probability so that the damaged guanine becomes a mutagenic site. Methylation of *MGMT* gene promoter has been associated with loss or decrease of *MGMT* expression in tumor tissues of various cancers, including lung tumors [6-8].

Taken together, methylation of *MGMT* gene has been considered as potentially useful candidate biomarker for early detection of lung cancer. Many studies had also shown that methylation of the gene can be found in clinical samples, such as tissues, serum and bronchoalveolar lavage fluid (BALF) of NSCLC [9-11].

The purpose of this study was to understand the difference of the prevalence of aberrant promoter methylation of *MGMT* in NSCLC tissue from control. We conducted a meta-analysis using available data in the literature on the basis of *MGMT* promoter methylation and NSCLC to better identify the association between *MGMT* promoter methylation and NSCLC.

## Materials and Methods

### Selection criteria and study search

We searched the electronic databases online including Pubmed, EMBASE, Ovid, ISI Web of Science, Elsevier and CNKI database, using the search terms “*MGMT*”, “NSCLC” and “methylation”. The following search strategy was performed in Pubmed “NSCLC” (MESH), “methylation” and “*MGMT* or O^6^-methylguanine-DNA methyl-transferase” to collect eligible articles. The search was limited to articles published in English and Chinese. Similar searches were performed in other databases. The search was updated until June 1, 2013.

Studies selected had to meet the following criteria: (a) The study was about *MGMT* methylation and NSCLC. (b) The authors offered a measure of the association either as an effect estimate with 95% CI and OR, or sufficient data in the original article to calculate it. (c) *MGTM* methylation status was examined using methylation-specific PCR (MSP) or real-time quantitative MSP (Q-MSP). (d) Specimens of NSCLC were surgically respected primary tumor sample and the styles of control were composed of plasma or non-cancerous lung tissues (NLT) including autologous control and heterogeneous control.

**Figure 1:**
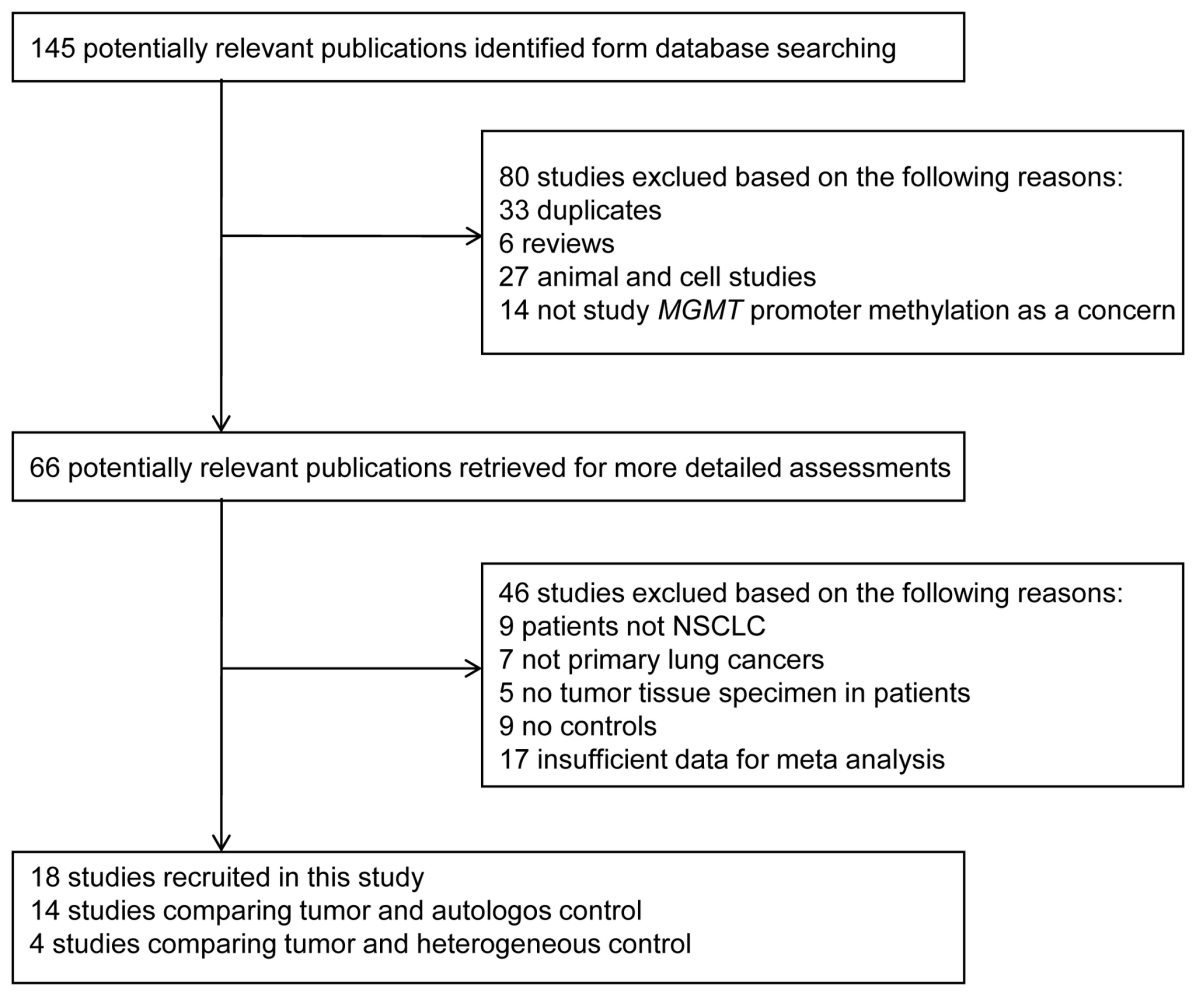
Flow diagram of the stepwise selection from associated studies.

Studies were excluded according the following criteria: (a) The study did not provide control information. (b) The articles were repetitively reported, (c) The articles researched chromate lung cancer, brain metastases of lung cancers and malignant pleural mesothelioma [7,12,13]. Firstly, we evaluated whether a study met the inclusion criteria by title and abstract of initial searching articles. Then all the potentially relevant articles were evaluated by accessing full-text paper. The selection procedure of studies was illustrated in statement flow chart (Figure 1).

### Abstraction of Data and Quality Assessment

All the data were independently abstracted by three authors (Changmei Gu, Cheng Lu, and Hao Shi) with the use of standardized data-extraction forms. For each study, the following characteristics were extracted: first author’s name, year of publication, the study country, ethnicity, age, sample size, type of control, the method for methylating detection, and status of *MGMT* methylation were collected using standard data extraction forms. If there were disagreements, we discussed with Meixia Lu and Jiachun Lu repeatedly, until a consensus. To ensure the transparent and complete reporting of this meta-analysis, we designed and checked selected studies according to the Preferred Reporting Items for Systematic Reviews and Meta-Analyses (PRISMA) [14] statement.

### Statistical Methods

The ORs and 95% CIs were abstracted or calculated to evaluate the strength of the association between *MGMT* promoter methylation and NSCLC risk. Summary of OR was acquired from all studies or calculated with the data in selected studies.

**Figure 2:**
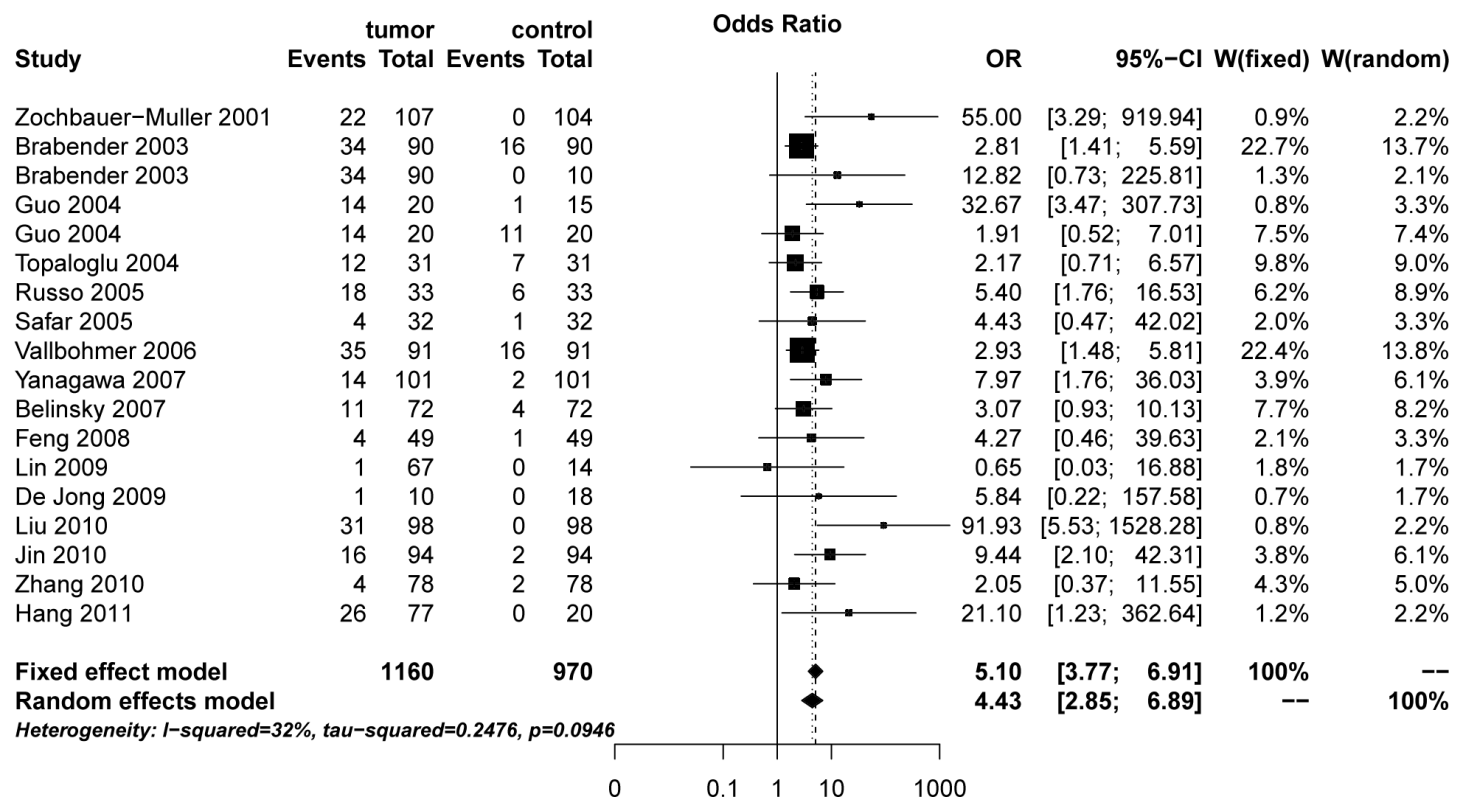
Forest plot of MGMT methylation in tumor tissue verse control group between *MGMT* promoter methylation and NSCLC.

We, then, investigated between-study heterogeneity by using Cochran’s Q test with a significance level of *P* value less than 0.1 and heterogeneity was also examined using *I* ^2^ statistic [15], which is a quantitative measure of inconsistency across studies. If *I* ^2^ > 50% or *P* < 0.1 is considered as a measure of severe heterogeneity, then the random-effects model was used to calculate summary OR according to the Der-Simonian Laird method; otherwise, the fixed-effects model (Matel–Haenszel method) was applied[16]. τ^2^ was used to determine how much heterogeneity was explained by subgroup differences. The meta-regression was performed to explore the source of heterogeneity based on ethnicity (Asian and Caucasian), publication year, style of control (autogenous or heterogeneous), method (MSP or Q-MSP), and sample size. Subgroup analyses were performed according to ethnicity (Asian and Caucasian), control style (autogenous or heterogeneous) and method of methylation detection (MSP or Q-MSP) in consideration of the source of heterogeneity. Sensitivity analyses were also performed to assess the contributions of each study on the overall result by omitting one study at a time. The funnel plot asymmetry was used to evaluate the evidence of publication bias. Peter’s test was applied to quantitatively evaluate the evidence for publication bias [17]. Fail-safe number (N_fs_) adopted by Rosenthal [28] is considered to be a useful indicator of leaning [18]. The meta-trim method was used to re-estimate the effect size when there was possible bias. All *P* values are two-tailed with a significant level at 0.05. In forest plot displayed in Figure 2, the size of the box for each study was inversely proportional to the variance of the log relative risk, and the horizontal lines represent 95% CI.

All statistical analyses were conducted by using the Meta package (version 2.2-1) in R (version 3.0, http://www.r-project.org/).

## Results

### Study Characteristics

The electronic search found that 145 potentially relevant studies were initially identified. These studies were further screened based on inclusion and exclusion criteria. A total of 18 studies (2001-2011) were included in the analysis (Figure 1). In these studies, 6 studies were conducted in Asia (1 in Japan and 5 in China) and the remaining 11 were in the USA and 1 in the Netherlands.

Among the 18 retrieved studies, 14 studies used methylation-specific polymerase chain reaction (MSP) and 4 studies used real-time quantitative MSP (Q-MSP) to explore *MGMT* methylation in NSCLC tissue and control. There were two control styles, including autogenous control (the tissues from the patients themselves) and heterogeneous control (i.e Plasma, tissue, and bronchoalveolar lavage fluid from other individuals). The main characteristics of these studies were presented in Table 1.

**Table 1:** Characteristics of studies included in this meta-analysis.

					Gender			Patients	Control	Control	Control	
Study	Year	Country		Ethnicity	M/F	Age (x)	Method	M+/N	M+/N	style	source	
Zochbauer[19]	2001	USA	Caucasian		76/31	28-81	MSP	22/107	0/104	A	tissue
Brabender[20]	2003	USA	Caucasian		68/22	63.3	Q-MSP	34/90	16/90	A	tissue
Brabender[20]	2003	USA	Caucasian		NA	NR	Q-MSP	34/90	0/10	H	tissue
Guo[9]	2004	USA	Caucasian		NA	42-83	MSP	14/20	1/15	A	tissue
Guo[9]	2004	USA	Caucasian		NA	42-83	MSP	14/20	11/20	A	BALF
Topaloglu[21]	2004	USA	Caucasian		NA	NA	Q-MSP	12/31	7/31	A	tissue
Russo[22]	2005	USA	Caucasian		NA	NA	MSP	18/33	6/33	A	serum
Safar[23]	2005	USA	Caucasian		NA	67	MSP	4/32	1/32	A	tissue
Vallbohmer[24]	2006	USA	Caucasian		69/22	63	MSP	35/91	16/91	A	tissue
Yanagawa[25]	2007	Japan	Asian		72/29	39-86	MSP	14/101	2/101	A	tissue
Belinsky[26]	2007	USA	Caucasian		49/23	37-80	MSP	11/72	4/72	A	serum
Feng[27]	2008	USA	Caucasian		24/23	64.3	MSP	4/49	1/49	A	tissue
Lin[28]	2009	China	Asian		NA	NA	MSP	1/67	0/14	H	tissue
De Jong[29]	2009	NE	Caucasian		NA	40-70	Q-MSP	1/10	0/18	H	tissue
Liu[30]	2010	China	Asian		58/40	32-68	MSP	31/98	0/98	H	tissue
Jin[31]	2010	China	Asian		83/11	32-75	MSP	16/94	2/94	A	tissue
Zhang[32]	2011	China	Asian		58/20	35-80	MSP	4/78	2/78	A	tissue
Hang[33]	2011	China	Asian		64/32	33-70	MSP	26/77	0/20	H	tissue

Abbreviation: A: Autologos control(the control from the NSCLC themselves); BALF: bronchoalveolar lavage fluid; H: Heterogeneous control(the control from other individuals, including serum, bronchoalveolar lavage or tissue); M+: The number of methylation; MSP: methylation-specific polymerase chain reaction; N: number of total; NA: not applicable; NE: Netherlands; Q-MSP: real-time quantitative methylation-specific polymerase chain reaction

There are 1, 160 NSCLC tumor tissues and 970 controls. The frequencies of *MGMT* promote methylation ranged from 1.5% to 70.0% (median 26.1%) in NSCLC tissue and 0.0% to 55.0% (median 2.4%) in non-cancerous control, respectively, which indicated the methylation frequency in cancer tissue was much higher than that in the control group. Under the random-effects model, the pooled OR of *MGMT* methylation in NSCLC tissue was 4.43 (95% CI: 2.85, 6.89), in comparison with control group (Figure 2).

### Meta-regression and subgroup analyses

Considering the existence of heterogeneity in the meta-analysis (*I* ^2^=32.0%, *P*= 0. 095), the meta-regression was performed for finding the source of heterogeneity. The restricted maximum likelihood modification was used to estimate between study variances. As the restriction to access raw data, we assumed the source of heterogeneity may appear from the year of publication, ethnicity, type of control, detection method and sample size. The results showed that the sources of the heterogeneity were control style (*P* = 0.073) and detection method (*P* = 0.094). Other factors such as sample size, year of publication, and ethnicity could not explain the heterogeneity (Table 2).

**Table 2:** Mixed-effects model of meta-regression analysis.

			95%CI		
Heterogeneity sources	Coefficients	*Z*	Lower	Upper	*P*
Year	-0.1964	-1.4009	-0.4712	0.0784	0.161
Control	1.4501	1.7915	-0.1364	3.0365	0.073
Ethnicity	0.9485	1.3741	-0.4044	2.3015	0.169
Method	-0.8260	-1.6752	-1.7925	0.1404	0.094
Sample size	0.0014	0.4581	-0.0046	0.0075	0.065

With these observations, we performed subgroup analyses based on control style and detection method. The OR in the heterogeneous control subgroup was 20.45 (95% CI: 5.83, 71.73; fixed-effects model), while it was 4.16 (95% CI: 3.02,5.72; fixed-effects model) in the autologous control subgroup. *I* ^2^ changed to 16.6% and 34.0% in those subgroups, respectively, compared with 32% of the total. Stratification by control style showed that the OR in the heterogeneous control subgroup was higher than that in the autologous control subgroup. After stratification by detection method, the OR was 5.31 (95% CI: 3.00, 9.41; random-effects model) in MSP subgroup and 3.06 (95% CI: 1.75, 5.33; fixed-effects model) in Q-MSP subgroup (Table 3).

**Table 3:** Subgroup analysis of the association between *MGMT* promoter methylation and NSCLC.

	Tumor		Control		M-H pooled OR^b^	D+L pooled OR^a^	Heterogeneity		
Group	M+	N	M+	N	OR (95%CI)	OR (95%CI)	*I* ^2^ (%)	*P*	τ^2^
Control group									
Auologous	202	818	69	810	4.16 (3.02, 5.72)	3.74 (2.54, 5.51)	16.6	0.276	0.080
Heterogeneous	93	342	0	160	20.45 (5.83, 71.73)	11.18 (2.14, 58.35)	34.0	0.194	1.208
Ethnicity									
Asian	92	515	6	405	10.96 (5.20, 23.14)	7.36 (2.33, 23.19)	45.1	0.105	0.8807
Caucasian	203	645	63	565	4.00 (2.85, 5.60)	3.49 (2.37, 5.14)	8.9	0.358	0.0418
Method									
MSP	214	939	46	821	6.17 (4.28, 8.91)	5.31 (3.00, 9.41)	40.2	0.060	0.4155
Q-MSP	81	221	23	149	3.06 (1.75, 5.33)	2.84 (1.62, 5.00)	0.0	0.674	<0.0001
Total	295	1160	69	970	5.10 (3.77, 6.91)	4.43 (2.85, 6.89)	32.0	0.095	0.2476

D+L pooled OR^a^: the result from random-effects model;M-H pooled OR^b^: the result from fixed-effects model.

### Sensitivity analysis

**Figure 3:**
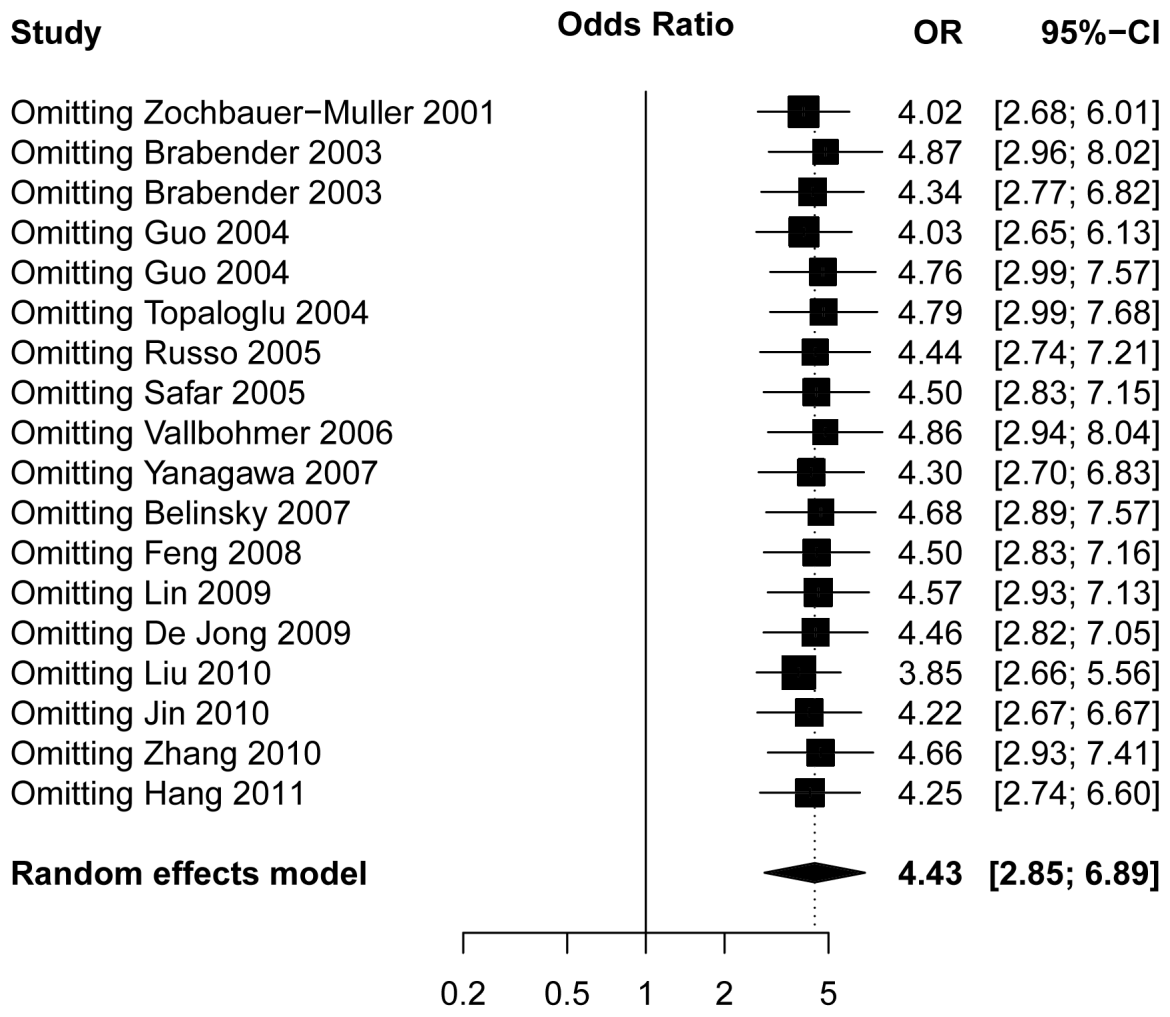
Sensitivity analysis by omitting a single study at a time on the overall effect.

To determine the effects of omitting a single study at a time on the overall effect, the sensitivity analysis was performed. Omission of a single study changed the overall OR from 3.85 (95% CI: 2.66, 5.56) to 4.87 (95% CI: 2.96, 8.02) using the random-effects model, which demonstrated that no single sensitivity analysis existed (Figure 3).

### Publication bias

**Figure 4:**
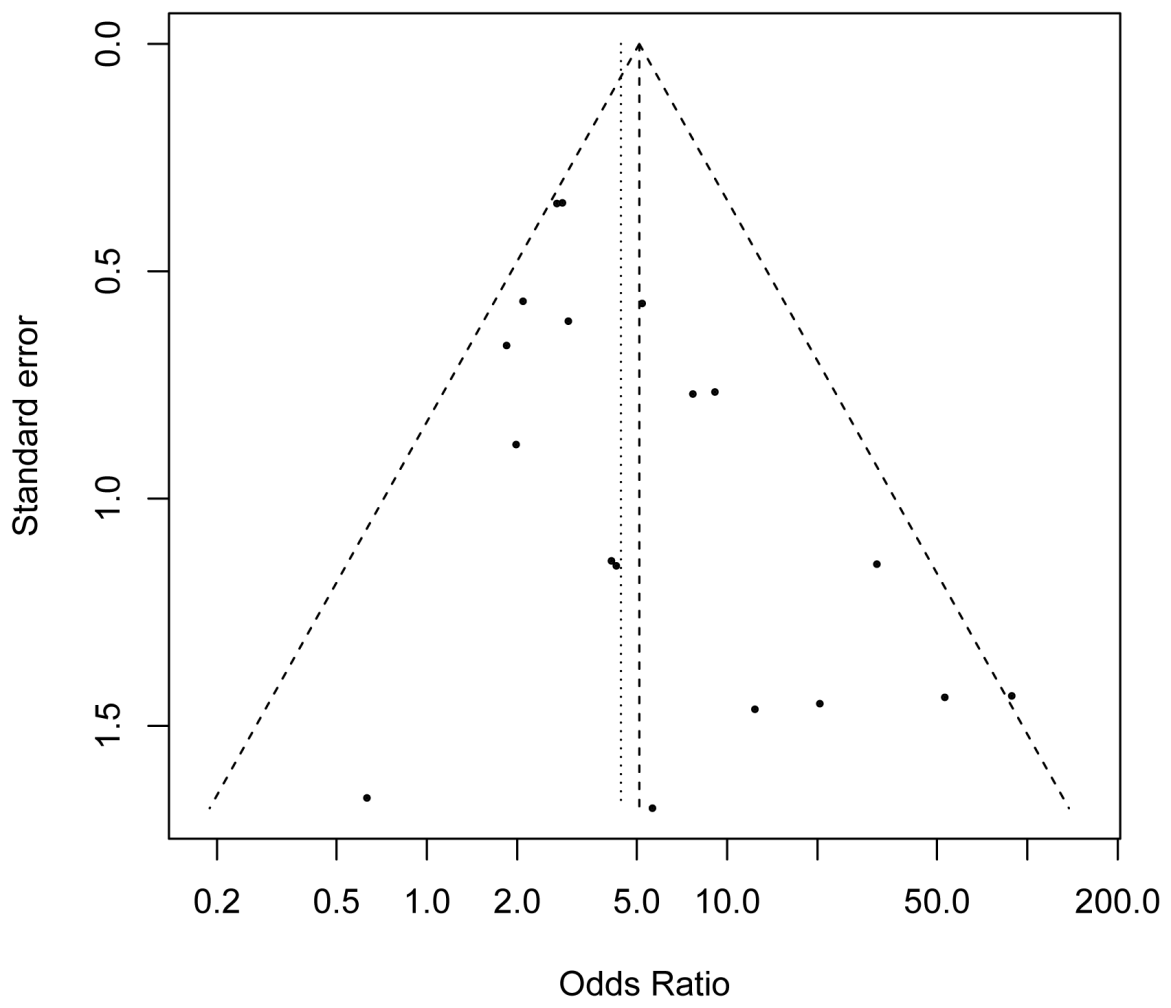
Funnel plot for assessment of publication bias. **Each hollow point represents a separate study for the indicated association**. The area of the hollow point reflects the weight (inverse of the variance). Horizontal line stands for the mean magnitude of the effect.

A funnel plot of methylation status of tumor versus control showed that there were two studies exceed the 95% confidence limits as shown in Figure 4. However, no publication bias was detected by using Peter’s test (*P* = 0.624). We also used a fail-safe number (N_fs_) to assess the efficacy of meta-analysis (*Z* = 45.63, N_fs0. 05_ = 756.12, N_fs0. 01_ = 365.52), which indicated a slight publication bias in the meta-analysis.

## Discussion

Aberrant methylation of promoter regions in DNA repair genes is a key event in the formation and progression of cancer. It was concluded that genes methylation was potentially a new generation of cancer biomarker [34]. Epigenetic change of CpG islands in the gene promoter region is an important reason for gene dysfunction [35], which can lead to the transcription of the gene down or stops [36].

*MGMT* promoter methylation is very common in the primary NSCLC, which has been reported in some studies. However, the results about the association between the status of *MGMT* methylation and NSCLC were inconsistent. The OR values fluctuated from 0.65 [28] to 114.98 [24], so we performed this meta-analysis to identify the association between *MGMT* promoter methylation and NSCLC.

A total of 18 studies including 1, 160 tumor tissues and 970 controls were involved in the meta-analysis. The frequencies of *MGMT* promote methylation ranged from 1.5% to 70.0% (median; 26.1%) in NSCLC tissue and 0.0% to 55.0% (median; 2.4%) in non-cancerous control, respectively. The findings indicated that the methylation frequency in cancer tissue was much higher than that in the control group. *MGMT* methylation had an increased risk in tumor tissue (OR = 4.43; 95% CI: 2.85, 6.89) in comparison with non-cancerous samples including plasma, tissue, and bronchoalveolar lavage fluid. This finding was consistent with other studies [10,25]. Furthermore, subgroup analysis of control style showed that an OR was 20.45 (95% CI: 5.83, 71.73; fixed-effects model) in the heterogeneous control subgroup verse 4.16 (95% CI: 3.02, 5.72; random-effects model) in the autologous tissues subgroup, indicating that the OR of methylation in heterogeneous control group was higher than that in the autologous control group. It suggested that *MGMT* gene promoter methylation was a frequent event in NSCLC, but it rarely happened in the non-tumor group. Stratification analysis by different methods of methylation detection showed that the OR for the MSP subgroup was higher than that for Q-MSP subgroup.

Sensitivity analysis was performed to determine the effects when omitting a single study at a time on the overall effect. The analytic results demonstrated that no single study could affect the summarized OR. In addition, the shape of the funnel plot did not show any evidence of funnel plot asymmetry and no publication bias was detected.

The current meta-analysis has some limitations. Firstly, selection bias is inevitable due to the strategy restricted to articles published in English or Chinese. Secondly, although the total sample sizes in the meta-analysis were not more than 1, 000, the results maybe have still no vigorous power. Thirdly, we did not study the methylation status in histological subtypes, smoking and different clinical stages because of limitation of insufficient raw materials.

In conclusion, *MGMT* gene is an important DNA repair gene on maintaining the integrity of the genome. Aberrant *MGMT* promoter methylation may be associated with the occurrence and development of NSCLC. This meta-analysis identified a strong association between *MGMT* promoter methylation and NSCLC. Prospective studies should be required to confirm the results in the future.

## Supporting Information

Checklist S1 **PRISMA Checklist.**
(DOCX)

## References

[1] Siegel R, Naishadham D, Jemal A (2013) Cancer statistics, 2013. CA Cancer J Clin 63: 11-30. doi:10.3322/caac.21166. PubMed: 23335087. 23335087 10.3322/caac.21166

[2] Esteller M (2008) Epigenetics in cancer. N Engl J Med 358: 1148-1159. doi:10.1056/NEJMra072067. PubMed: 18337604. 18337604 10.1056/NEJMra072067

[3] Lu J, Liu R, Li B, Qiu X, Li Q et al. (2003) MGMT expression and its relationship with efficacy of chemotherapy and prognosis in patients with non-small cell lung cancer. Chin J Lung Cancer 6: 63-66. 10.3779/j.issn.1009-3419.2003.01.16 21262152

[4] Ekim M, Caner V, Buyukpinarbasili N, Tepeli E, Elmas L et al. (2010) Determination of O6-methylguanine DNA methyltransferase promoter methylation pattern in non-small cell lung cancer. Clin Genet 78: 65-67. 10.1089/gtmb.2010.0211 21288129

[5] Belinsky SA, Klinge DM, Liechty KC, March TH, Kang T et al. (2004) Plutonium targets the p16 gene for inactivation by promoter hypermethylation in human lung adenocarcinoma. Carcinogenesis 25: 1063-1067. doi:10.1093/carcin/bgh096. PubMed: 14742312. 14742312 10.1093/carcin/bgh096

[6] Zou XP, Zhang B, Zhang XQ, Chen M, Cao J et al. (2009) Promoter hypermethylation of multiple genes in early gastric adenocarcinoma and precancerous lesions. Hum Pathol 40: 1534-1542. doi:10.1016/j.humpath.2009.01.029. PubMed: 19695681. 19695681 10.1016/j.humpath.2009.01.029

[7] Fujii M, Fujimoto N, Hiraki A, Gemba K, Aoe K et al. (2012) Aberrant DNA methylation profile in pleural fluid for differential diagnosis of malignant pleural mesothelioma. Cancer Sci 103: 510-514. doi:10.1111/j.1349-7006.2011.02180.x. PubMed: 22146010. 22146010 10.1111/j.1349-7006.2011.02180.x PMC7713611

[8] Esteller M, Sanchez-Cespedes M, Rosell R, Sidransky D, Baylin SB et al. (1999) Detection of aberrant promoter hypermethylation of tumor suppressor genes in serum DNA from non-small cell lung cancer patients. Cancer Res 59: 67-70. PubMed: 9892187. 9892187

[9] Guo M, House MG, Hooker C, Han Y, Heath E et al. (2004) Promoter hypermethylation of resected bronchial margins: a field defect of changes? Clin Cancer Res 10: 5131-5136. doi:10.1158/1078-0432.CCR-03-0763. PubMed: 15297416. 15297416 10.1158/1078-0432.CCR-03-0763

[10] Citron M, Schoenhaus M, Graver M, Hoffman M, Lewis M et al. (1993) O6-methylguanine-DNA methyltransferase in human normal and malignant lung tissues. Cancer Invest 11: 258-263. doi:10.3109/07357909309024850. PubMed: 8485648. 8485648 10.3109/07357909309024850

[11] Higgins JP (2008) Commentary: Heterogeneity in meta-analysis should be expected and appropriately quantified. Int J Epidemiol 37: 1158-1160. doi:10.1093/ije/dyn204. PubMed: 18832388. 18832388 10.1093/ije/dyn204

[12] Ali AH, Kondo K, Namura T, Senba Y, Takizawa H et al. (2011) Aberrant DNA methylation of some tumor suppressor genes in lung cancers from workers with chromate exposure. Mol Carcinog 50: 89-99. doi:10.1002/mc.20697. PubMed: 21229606. 21229606 10.1002/mc.20697

[13] Wu PF, Kuo KT, Kuo LT, Lin YT, Lee WC et al. (2010) O(6)-Methylguanine-DNA methyltransferase expression and prognostic value in brain metastases of lung cancers. Lung Cancer 68: 484-490. doi:10.1016/j.lungcan.2009.08.010. PubMed: 19740564. 19740564 10.1016/j.lungcan.2009.08.010

[14] Moher D, Liberati A, Tetzlaff J, Altman DG (2009) Preferred reporting items for systematic reviews and meta-analyses: the PRISMA statement. BMJ 151: 264-269. PMC3090117 21603045

[15] Higgins JP, Thompson SG, Deeks JJ, Altman DG (2003) Measuring inconsistency in meta-analyses. BMJ 327: 557-560. doi:10.1136/bmj.327.7414.557. PubMed: 12958120. 12958120 10.1136/bmj.327.7414.557 PMC192859

[16] Stuck AE, Rubenstein LZ, Wieland D (1998) Bias in meta-analysis detected by a simple, graphical test. Asymmetry detected in funnel plot was probably due to true heterogeneity. BMJ 316: 469-471. doi:10.1136/bmj.316.7129.469. PMC2665578 9492685

[17] Rücker G, Schwarzer G, Carpenter J (2008) Arcsine test for publication bias in meta-analyses with binary outcomes. Stat Med 27: 746-763. doi:10.1002/sim.2971. PubMed: 17592831. 17592831 10.1002/sim.2971

[18] Rosenthal R (1979) The file drawer problem and tolerance for null results. Psychol Bull 86: 638-641. doi:10.1037/0033-2909.86.3.638.

[19] Zöchbauer-Müller S, Fong KM, Virmani AK, Geradts J, Gazdar AF et al. (2001) Aberrant promoter methylation of multiple genes in non-small cell lung cancers. Cancer Res 61: 249-255. PubMed: 11196170. 11196170

[20] Brabender J, Usadel H, Metzger R, Schneider PM, Park J et al. (2003) Quantitative O(6)-methylguanine DNA methyltransferase methylation analysis in curatively resected non-small cell lung cancer: associations with clinical outcome. Clin Cancer Res 9: 223-227. PubMed: 12538473. 12538473

[21] Topaloglu O, Hoque MO, Tokumaru Y, Lee J, Ratovitski E et al. (2004) Detection of promoter hypermethylation of multiple genes in the tumor and bronchoalveolar lavage of patients with lung cancer. Clin Cancer Res 10: 2284-2288. doi:10.1158/1078-0432.CCR-1111-3. PubMed: 15073103. 15073103 10.1158/1078-0432.ccr-1111-3

[22] Russo AL, Thiagalingam A, Pan H, Califano J, Cheng KH et al. (2005) Differential DNA hypermethylation of critical genes mediates the stage-specific tobacco smoke-induced neoplastic progression of lung cancer. Clin Cancer Res 11: 2466-2470. doi:10.1158/1078-0432.CCR-04-1962. PubMed: 15814621. 15814621 10.1158/1078-0432.CCR-04-1962

[23] Safar AM, Spencer HR, Su X, Coffey M, Cooney CA et al. (2005) Methylation profiling of archived non-small cell lung cancer: a promising prognostic system. Clin Cancer Res 11: 4400-4405. doi:10.1158/1078-0432.CCR-04-2378. PubMed: 15958624. 15958624 10.1158/1078-0432.CCR-04-2378

[24] Vallböhmer D, Brabender J, Yang D, Schneider PM, Metzger R et al. (2006) DNA methyltransferases messenger RNA expression and aberrant methylation of CpG islands in non-small-cell lung cancer: association and prognostic value. Clin Lung Cancer 8: 39-44. doi:10.3816/CLC.2006.n.031. PubMed: 16870044. 16870044 10.3816/CLC.2006.n.031

[25] Yanagawa N, Tamura G, Oizumi H, Kanauchi N, Endoh M et al. (2007) Promoter hypermethylation of RASSF1A and RUNX3 genes as an independent prognostic prediction marker in surgically resected non-small cell lung cancers. Lung Cancer (Amst, Netherlands) 58: 131-138. doi:10.1016/j.lungcan.2007.05.011. PubMed: 17606310. 10.1016/j.lungcan.2007.05.011 17606310

[26] Belinsky SA, Grimes MJ, Casas E, Stidley CA, Franklin WA et al. (2007) Predicting gene promoter methylation in non-small-cell lung cancer by evaluating sputum and serum. Br J Cancer 96: 1278-1283. doi:10.1038/sj.bjc.6603721. PubMed: 17406356. 17406356 10.1038/sj.bjc.6603721 PMC2360148

[27] Feng Q, Hawes SE, Stern JE, Wiens L, Lu H et al. (2008) DNA methylation in tumor and matched normal tissues from non-small cell lung cancer patients. Cancer Epidemiol Biomarkers Prev 17: 645-654. doi:10.1158/1055-9965.EPI-07-2518. PubMed: 18349282. 18349282 10.1158/1055-9965.EPI-07-2518 PMC2798850

[28] Lin Q, Geng J, Ma K, Yu J, Sun J et al. (2009) RASSF1A, APC, ESR1, ABCB1 and HOXC9, but not p16INK4A, DAPK1, PTEN and MT1G genes were frequently methylated in the stage I non-small cell lung cancer in China. J Cancer Res Clin Oncol 135: 1675-1684. doi:10.1007/s00432-009-0614-4. PubMed: 19506903. 19506903 10.1007/s00432-009-0614-4 PMC11844779

[29] De Jong WK, Verpooten GF, Kramer H, Louwagie J, Groen HJ (2009) Promoter methylation primarily occurs in tumor cells of patients with non-small cell lung cancer. Anticancer Res 29: 363-369. PubMed: 19331174. 19331174

[30] Liu Y, Wang J, Miao L, Wu Y, Wu Y (2010) Promoter hyperthylation of MGMT gene and expression of K-ras in human NSCLC. J Medical FORUM 31: 1-4.

[31] Jin Y, Xu H, Zhang C, Zhang H, Xue S et al. (2010) Association of abnormal methylation of CpG islands in promoter domains of multiple tumor suppressor genes with non-small cell lung cancer. Chin J Clin Oncol 37: 1109-1114.

[32] Zhang Y, Wang R, Song H, Huang G, Yi J et al. (2011) Methylation of multiple genes as a candidate biomarker in non-small cell lung cancer. Cancer Lett 303: 21-28. doi:10.1016/j.canlet.2010.12.011. PubMed: 21255913. 21255913 10.1016/j.canlet.2010.12.011

[33] Hang C, Tao Z, Xiao H, Zhang X, Xue Y (2011) Investigation on the relationgship between the promoter hypermethylationof MGMTand clinicopathologic feature inNSCLC. Chin J Laboratory Diagn 15: 81-82.

[34] Kim YT, Lee SH, Sung SW, Kim JH (2005) Can aberrant promoter hypermethylation of CpG islands predict the clinical outcome of non-small cell lung cancer after curative resection? Ann Thorac Surg 79: 1180-1188. doi:10.1016/j.athoracsur.2004.09.060. PubMed: 15797047. 15797047 10.1016/j.athoracsur.2004.09.060

[35] Kim DS, Cha SI, Lee JH, Lee YM, Choi JE et al. (2007) Aberrant DNA methylation profiles of non-small cell lung cancers in a Korean population. Lung Cancer 58: 1-6. doi:10.1016/j.lungcan.2007.04.008. PubMed: 17532092. 17532092 10.1016/j.lungcan.2007.04.008

[36] Citron M, Decker R, Chen S, Schneider S, Graver M et al. (1991) O6-methylguanine-DNA methyltransferase in human normal and tumor tissue from brain, lung, and ovary. Cancer Res 51: 4131-4134. PubMed: 1868433. 1868433

